# Simulation-based architecture of a stable large-area $JDBD$ atmospheric plasma source

**DOI:** 10.1038/s41598-023-29143-5

**Published:** 2023-02-03

**Authors:** M. V. Roshan, S. Razaghi, A. Singh

**Affiliations:** 1 Physics Department, University of Malaya, Jln Professor Aziz, 50603 Kuala Lumpur, Wilayah Persekutuan Kuala Lumpur Malaysia; 2 Physics Department, Amirkabir University of Technology, Tehran, 15875-4413 Iran; 3 Faculty of Engineering, INTI International University, 71800 Putra Nilai, Malaysia

**Keywords:** Plasma physics, Biophysics

## Abstract

Unified jet-DBD design, $JDBD$, proposed in this work presents large-scale plasma in an unbounded region of atmospheric air, without any need for the flow of gas, offering efficient exposure to sizable and complex objects. This is a simulation-based architecture for stable non-thermal plasma source with notable experimental results. $JDBD$ geometry optimizes the electric field and charge distribution for a diffuse discharge in the steady air by a key design parameter of $efficient \; insulation$. Teflon insulator with a thickness ${d}_{Tf}\ge 10 \; \text{mm}$ imposes an intense and uniform electric field shaped up at the open area in front of the device and generates radially/axially expanded plasma jet. In the $JDBD$, phase shift increases by ${I}_{rms}$ and the plasma generates more power than the classical plasma jet. Two distinct states of $JDBD$ operation indicate the mode-swap at $0.8\; \text{mA}$ and power dissipation. In the reactive $JDBD$ scheme even small changes in the phase angle effectively improves the electric power.

## Introduction

Plasma jet produces non-thermal plasma propagating away from the confined electrode assembly into the atmospheric air and provides reactive species like $ROS \left(O, OH, {O}_{2}\right), RNS (NO, N{O}_{2})$, hydroxyl radical, $OH$, and hydrogen peroxide, ${H}_{2}{O}_{2}$, with both medical and industrial implications^1^. Plasma components including $UV$ photons, electric fields, ionic and reactive species enable a large range of synergistic mechanisms and it is a promising tool for medical treatments such as biofilm sterilization, and materials processing including surface modification and thin-film deposition^2^. Plasma sustains the biofilm disinfection protocols including safety, efficiency, and cost-effectiveness^3^.

Electric discharge is initiated in a neutral gas by setting the electrons to be free using some procedures such as external photons. Such free electrons are accelerated subsequently by an electric field, which is required to be present in the medium^4^. The electric potential enhances the fraction of electron–ion pairs subjected to the electric field to the point where the current is saturated. Beyond saturation potential, the electric current rises up exponentially by increasing the voltage. At this stage, the intense electric field sets the primary electrons, which are small and have higher mean free path compared with ions, to gain required energy to ionize the neutral atoms in the gas on their way toward anode and the secondary electrons are produced. Higher electric field makes secondary electrons to further ionize the neutrals and supply the electron avalanche. The region where current was increasing exponentially is called the Townsend discharge. Prior to the electrical gas breakdown, corona discharges occur in the regions where the electric field is strong, such as sharp points, in the so-called Townsend dark discharge regime. Glow discharge occurs at higher electric currents where visible light is emitted. At a low electric field where the corona discharge is active, the filamentary configuration is formed which is referred to as streamers^5^.

Extra secondary electrons emitted from the cathode due to the ion/photon impact lead to the electric breakdown in the gas. At the breakdown phase the electric current rises up by a few orders of magnitude, and the gas state moves into the natural glow discharge regime.

Atmospheric plasma sources, such as DBD, are equipped with a barrier-like configuration to produce the plasma, which is confined geometrically to the inter-electrodes area. Alternatively, in the plasma jet configuration the plasma is blown away to an unbound area. Electric field is increased by the applied potential, and higher frequency results in larger input power to the discharge. However, the optimum frequency related to the suitable applied power needs to be characterized, since frequency beyond a certain limit does not necessarily improve the plasma attributes, as reported in Ref^6^ for $N{O}_{x}$ treatment. The electron emission efficiency, electric permittivity coefficient, and electrical capacitance are the main parameters of the electrode and dielectric materials to be optimized.

DBD generates extensive plasma except that is bound in the inter-electrode spacing or attached to the single electrode. In the plasma jet, the gas flowing through the device extends the plasma as an efflux into the ambient air with mm-*scale* sharp configuration^7–15^. Unified jet-DBD design, $JDBD$, as proposed in this work, presents large-scale plasma in an unbounded region of atmospheric air offering efficient exposure to sizable and complex objects. This is a simulation-based design for an efficient and stable atmospheric air plasma source with notable experimental results.

### *JDBD* model

Due to the electric field and electrode configuration, the plasma plume is forced out of the tube into the open air, yet a common drawback in the plasma jet is the relatively small dimension of the discharge demanding noble gas equipped with a flow controller. Inherent in every AC voltage cycle is a controlling plasma-off that reduces the charge in the central electrode tip neighborhood and subsequently the metastable atoms are blown away leading to path preferences. The recombination process generates high density metastable atoms accompanied with an efficient ionization if combined well with the seed electrons^16^. Radial component of the electric field guides the electron drift velocity and drives the discharge propagation and expansion into the surrounding air. Thus, higher electron density in this area yields homogenous radial discharge.

**Figure 1:**
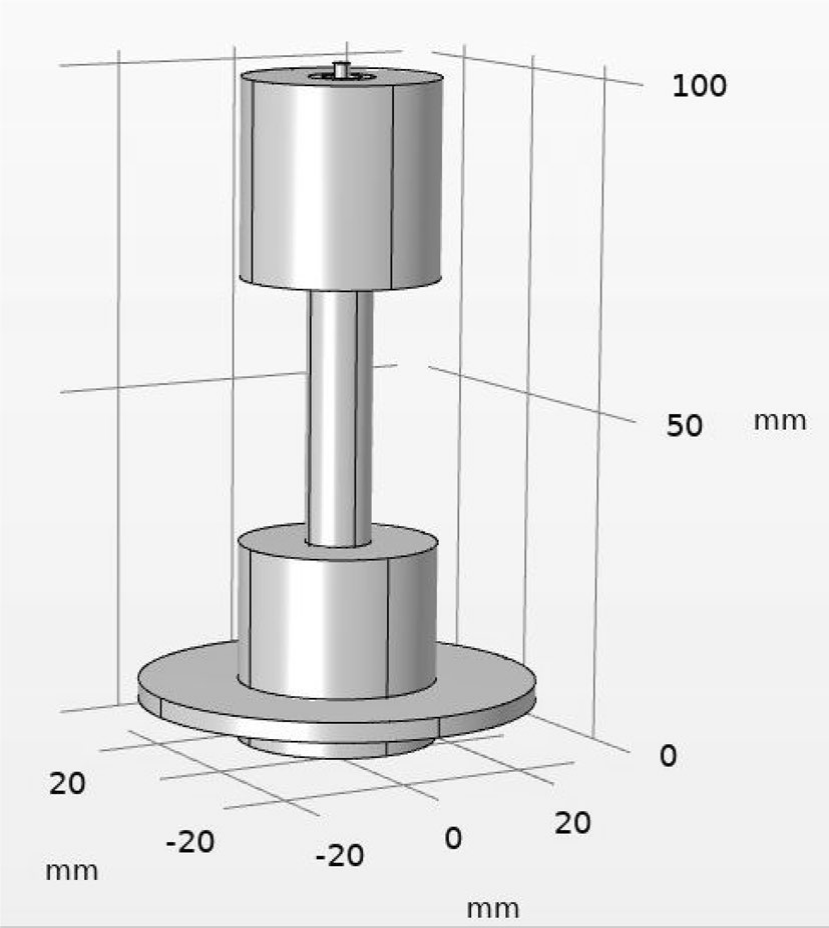
Three-D geometry of simulated $JDBD$ (COMSOL Multiphysics).

$JDBD$ geometry, Fig. 1, optimizes the electric field and charge distribution for diffuse and stable discharges in the steady atmospheric air without any need for the flow of gas, by a key design parameter of “$efficient \; insulation$” being a major factor. For the plasma jet to be created in the atmospheric air, the discharge guidance is sustained by the axial and radial electric field. Covering the glass tube, within which the central electrode is located, with an expanded polyethene enhances the inter-electrode spacing and changes the electric properties of the discharge. The electric field within polyethylene falls off faster than air: $\frac{dE}{dx}=\frac{1}{\varepsilon {\varepsilon }_{0}}\rho \left(x\right)$.

$Central \; objective\;  in \; JDBD \; modeling$ is to solve the related equations in a way that the plasma jet is generated in front of/outside the device. Besides, the plasma chemistry is defined to take into account both surface interactions occurring in the wall in addition to the volume interactions. Gas temperature (293 K) and air number density is constant. The equation of drift diffusion is solved to compute the electron density:

1 $$\frac{\partial {n}_{e}}{\partial t}+\nabla \cdot {{\varvec{\Gamma}}}_{e}={S}_{e}$$

2 $${{\varvec{\Gamma}}}_{e}=-\left({\mu }_{e}{\varvec{E}}{n}_{e}+{D}_{e}\nabla {n}_{e}\right)$$

where ${{\varvec{\Gamma}}}_{e}$ is the flux density, ${S}_{e}$ is the net source term determined by the reactions occuring in the discharge which is proportional to the neutrals number density and rate coefficient, ${\varvec{E}}$ is the electric field calculated from the Poisson’s equation, and ${D}_{e}$ electron diffusion coefficient which is defined by electron temperature ${T}_{e}$ and mobility ${\mu }_{e}$ through ${D}_{e}={T}_{e} \cdot {\mu }_{e}$ .

Mass fraction, ${w}_{k}$, of non-electron species, consisting of ions and neutrals, was determined by:

3 $$\rho \frac{\partial }{\partial t}\left({w}_{k}\right)+\rho (\mathbf{u} \cdot \nabla ){w}_{k}={\nabla \cdot {\mathbf{j}}_{k}+R}_{k}$$

where diffusive flux vector and rate expression of the species $k$ are represented by ${\mathbf{j}}_{k}$ and ${\mathrm{R}}_{k}$. The space charge density based on the plasma chemistry for charge ${\mathrm{Z}}_{k}$ and number density ${\mathrm{n}}_{k}$ is given by:

4 $$\rho =q\left[\sum_{k=1}^{N}{Z}_{k}{n}_{k}-{n}_{e}\right]$$

Constitutive momentum equation of a continuous fluid, Navier–Stokes, models the plasma flow and the chemistry governs the space charge density.

**Figure 2:**
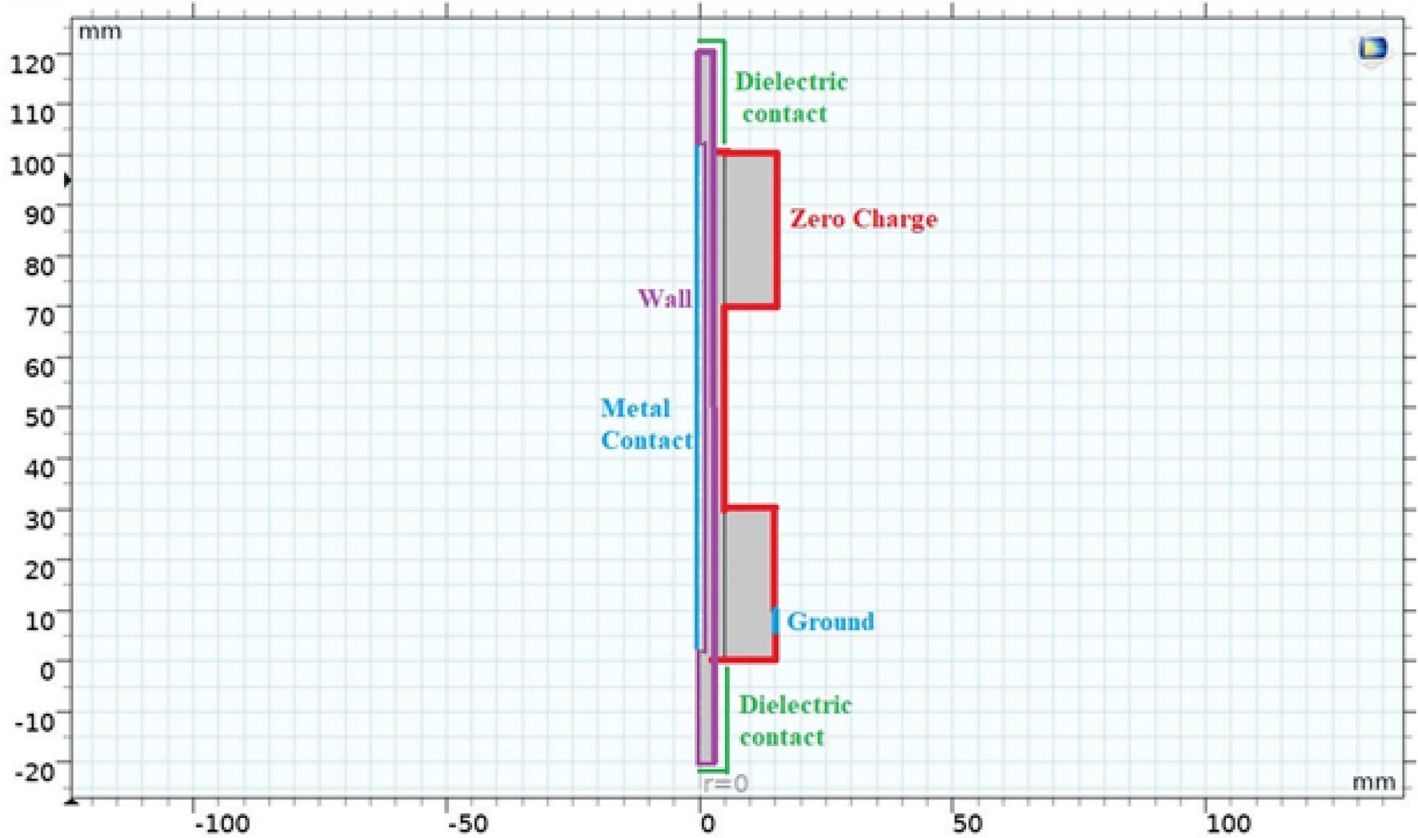
Model’s boundary definition.

Secondary electron emission within the walls imposes definitive boundary conditions, Fig. 2, in the model whereas the ions are lost in the wall by surface reactions.

Multiple chemical reactions of $Oxygen$ and $Nitrogen$ are set in the dry air (humidity reduces the discharge voltage leading to the fast generation and propagation of the plasma) to characterize the plasma chemistry processes. Chemical reactions within the discharge volume include most of the important $Oxygen$ and $Nitrogen$ interactions in addition to the reactions in the wall, Table 1. In the table ${K}_{f} ({\text{m}}^{3}/\text{s})$ is rate coefficient, ${x}_{air}$ (dimensionless) is mole fraction of air, ${\varepsilon }_{avg} \; (\text{eV})$ is average electron energy, ${T}_{e} \; (\text{eV})$ is electron temperature, ${T}_{eg}$ (dimensionless) is electron temperature normalized to the gas temperature, ${T}_{0}$ (dimensionless) is gas temperature normalized to $300 \; \text{K}$, and ${T}_{g} \; (\text{K})$ is the gas temperature^17,18^.

**Table 1:** List of $Oxygen$ and $Nitrogen$ chemical reactions.

Reaction	Formula	Type	$\Delta \varepsilon \; (\text{eV})$	${K}_{f} ({\text{m}}^{3}/\text{s})$
1	e + N_2_ → 2e + N_2_^+^	Ionization	15.6	$f({x}_{air},{\varepsilon }_{avg})$
2	e + N → 2e + N^+^	Ionization	14.5	1 × 10^–14^ ${T}_{e}$^0.5^ $\mathrm{exp}(-14.5/{T}_{e})$
3	e + N_2_ → 2e + N + N^+^	Ionization	28	4.2 × 10^–16^ ${T}_{e}$^0.5^ $\mathrm{exp}(-28/{T}_{e})$
4	e + N^+^ → N	Attachment	–	3.5 × 10^–18^
5	e + N_2_^+^ → 2N	Attachment	–	2.8 × 10^–13^ ${T}_{eg}$^−0.5^
6	e + N_2_^+^ → N_2_	Attachment	–	4.8 × 10^–13^ ${T}_{eg}$^−0.5^
7	2e + N^+^ → N + e	Attachment	–	7 × 10^–32^ ${T}_{eg}$^−4.5^
8	e + N_2_ + N^+^ → N + N_2_	Attachment	–	6 × 10^–39^ ${T}_{eg}$^−2.5^
9	2e + N_2_^+^ → N_2_ + e	Attachment	–	7 × 10^–32^ ${T}_{eg}$^−4.5^
10	e + N_2_ + N_2_^+^ → 2N_2_	Attachment	–	6 × 10^–39^ ${T}_{eg}$^−1.5^
11	3N → N + N_2_	Reaction	–	3.31 × 10^–39^ ${T}_{0}$^−1.5^
12	2N + N_2_ → 2N_2_	Reaction	–	7.6 × 10^–46^ $\mathrm{exp}(500/{T}_{g})$
13	N + N_2_^+^ → N_2_ + N^+^	Reaction	–	2.4 × 10^–21^ ${T}_{g}$
14	N_2_ + N + N^+^ → N_2_ + N_2_^+^	Reaction	–	10^–41^
15	N_2_ + e → N_2_^−^	Attachment	–	$f({x}_{air},{\varepsilon }_{avg})$
16	2N_2_ + e → N_2_^−^ + N_2_	Attachment	–	$f({x}_{air},{\varepsilon }_{avg})$
17	N_2_^+^ + N_2_^−^ → 2N_2_	Reaction	–	12.044 × 10^12^
18	e + O_2_ → 2e + O_2_^+^	Ionization	12.06	$f({x}_{air},{\varepsilon }_{avg})$
19	e + O_2_^+^ → O + O	Attachment	− 6.91	7.762 × 10^–15^ ε_avg_^−1^
20	2e + O_2_^+^ → O_2_ + e	Attachment	–	7 × 10^–32^ ${T}_{eg}$^−4.5^
21	e + O_2_ + O_2_^+^ → 2O_2_	Attachment	–	2.49 × 10_–41_ ${T}_{eg}$^−1.5^
22	e + O^−^ → O + 2e	Attachment	2.98	5.47 × 10^–14^ ${T}_{e}$^0.324^ $\mathrm{exp}(-2.98/{T}_{e})$
23	e + O^+^_2_ → O_2_	Attachment	–	4 × 10^–18^
24	e + O^+^ → O	Attachment	–	4 × 10^–18^
25	2e + O^+^ → O + e	Attachment	–	7 × 10^–32^ ${T}_{eg}$^−4.5^
26	e + O → 2e + O^+^	Ionization	13.6	9 × 10^–14^ ${T}_{e}$^0.7^ $\mathrm{exp}(-13.6/{T}_{e})$
27	e + O_2_ → 2e + O + O^+^	Ionization	17	5.4 × 10^–16^ ${T}_{e}$^0.5^ $\mathrm{exp}(-17/{T}_{e})$
28	e + O_2_ → O^−^ + e + O^+^	Ionization	17	7.1 × 10^–17^ ${T}_{e}$^0.5^ $\mathrm{exp}(-17/{T}_{e})$
29	2O → O_2_	Reaction	–	9.26 × 10^–40^ ${T}_{0}$^−1^
30	2O_2_ → 2O + O_2_	Reaction	–	6.6 × 10^–15^ ${T}_{0}$^−1.5^ $\mathrm{exp}(-59000/{T}_{g})$
31	3O → O_2_ + O	Reaction	–	9.21 × 10^–46^ ${T}_{0}$^−0.63^
32	2O + O_2_ → 2O_2_	Reaction	–	2.56 × 10^–46^ ${T}_{0}$^−0.63^
33	O_2_^−^ + O_2_^+^ → 2O + O_2_	Reaction	–	10^–13^
34	O_2_^−^ + O_2_^+^ → 2O_2_	Reaction	–	10^–13^
35	O_2_ + O_2_^−^ → 2O_2_ + e	Reaction	–	2.7 × 10^–16^ ${T}_{0}$^0.5^ $\mathrm{exp}(-5590/{T}_{g})$
36	O_2_^−^ + O_2_ + O_2_^+^ → 3O_2_	Reaction	–	2 × 10^–37^ ${T}_{0}$^−2.5^
37	O^−^ + O_2_^+^ → 3O	Reaction	–	10^–13^
38	O^−^ + O_2_^+^ → O + O_2_	Reaction	–	1 × 10^–13^ ${T}_{0}$^−0.5^
39	O^−^ + O → O_2_ + e	Reaction	–	2 × 10^–16^ ${T}_{0}$^0.5^
40	O^−^ + O_2_ → O + O_2_^−^	Reaction	–	1.5 × 10^–18^
41	O_2_ + O^+^ → O + O_2_^+^	Reaction	–	2 × 10^–17^ ${T}_{0}$^−0.4^
42	O + O_2_ + O^+^ → O_2_ + O_2_^+^	Reaction	–	1 × 10^–41^ ${T}_{0}$^0.5^
43	O^−^ + O^+^ → 2O	Reaction	–	2.7 × 10^–13^ ${T}_{0}$^−0.5^
44	O_2_^−^ + O^+^ → O + O_2_	Reaction	–	2 × 10^–13^ ${T}_{0}$^−1^
45	O^−^ + O_2_ + O^+^ → O_2_ + 2O	Reaction	–	2 × 10^–37^ ${T}_{0}$^−2.5^
46	O^−^ + O_2_ + O^+^ → 2O_2_	Reaction	–	2 × 10^–37^ ${T}_{0}$^−2.5^
47	O_2_^−^ + O_2_ + O^+^ → 2O_2_ + O	Reaction	–	2 × 10^–37^ ${T}_{0}$^−2.5^

In order to converge the solution to the equations, maximum and minimum element size were set to be $0.1 \; \text{mm} \; and \; 2.16 \; \upmu {\text{m}}$, and time dependent solver with $0 \; to \; 500 \; \text{ns}$ range and $0.1\; \text{ns}$ step was determined. Total Number of degrees of freedom varied slightly for different Teflon thicknesses.

A set of electrostatics equation and Laminar flow of the plasma are solved simultaneously with initial values of velocity on the $z{\text{-}}axis$ and pressure defined as $1 \; \text{m/s}$ and 1 atm, respectively. Electric potential of the metal contact boundary conditions is set to $\phi =2.5\times {10}^{4}\mathrm{sin}\left(2\pi \left(2.3\times {10}^{4}\right)t\right)$. Dielectric constant for Teflon and glass are $2.1$ and $4.7$. The model starts off with ${10}^{10}{\text{m}}^{-3}$ seed electron density and initial mean electron energy of $4 \; \text{V}$ on top of $0 \; \text{V}$ being set for the electric potential. The solutions to the equations are converged by setting off the minimum and maximum element size $2\times {10}^{-3}\; \text{mm}$ and $0.1\; \text{mm}$ with a time step $0.1 \; \text{ns}$ and time interval $0\to 400 \; \text{ns}$.

**Figure 3:**
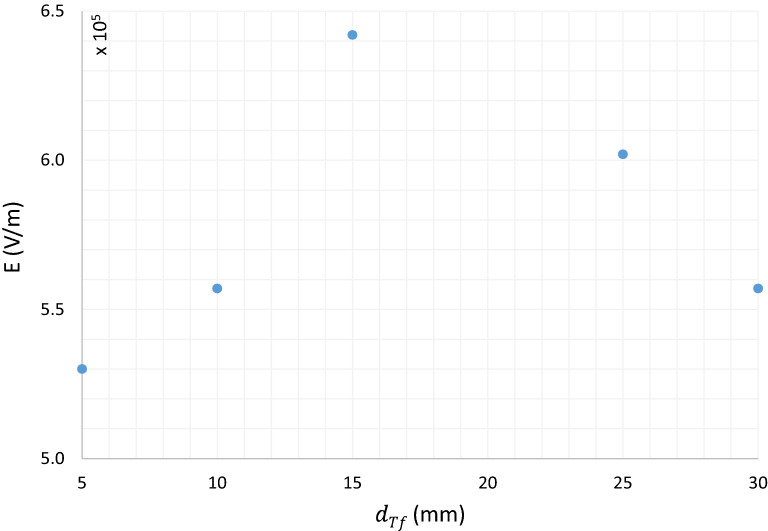
$JDBD$ electric field of the insulating parameter.

**Figure 4:**
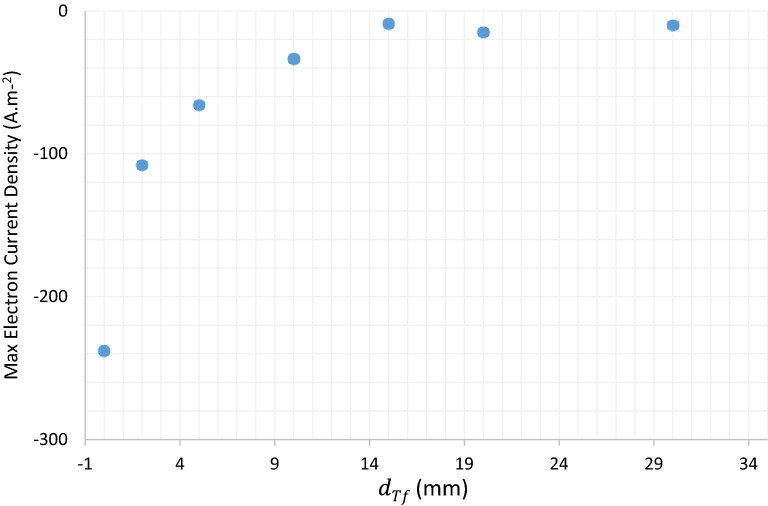
Electron current density at $t=150 \; \text{ns}$.

Detailed Analysis of $JDBD$ through simulation marks inherent challenges of fine gas chemistry and interactions coupled with the Laminar flow equations. Teflon insulator with a thickness of ${d}_{Tf}\ge 10\; \text{mm}$ imposes an intense and uniform electric field lines shaped up at the open area in front of the device and generates both radially and axially expanded plasma jet, Fig. 3 . The ordinate axis quantity is the maximum electric field norm at $t=150 \; \text{ns}$. The maximum electric field occurs near the lower end of the anode surface. The current density with a sudden steep, followed by a constant value run at peak electric fields, backs up the $JDBD$ simulated configuration, Fig. 4. Values are negative in this figure since the backward plasma propagation was oriented parallel to the positive component of the $Z{\text{-}}axis$.

**Figure 5:**
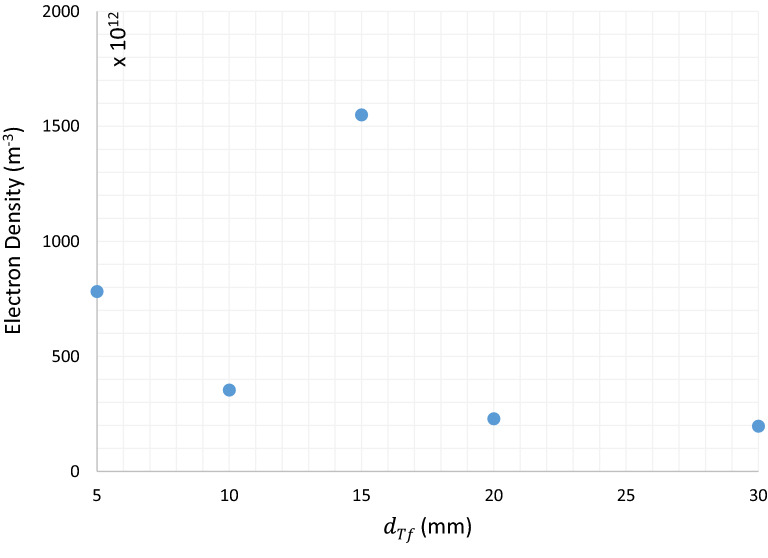
Mean electron density at $t=150\; \text{ns}$.

$JDBD$ simulation results in a mean electron temperature and maximum electron density, Fig. 5, of about $2.1\; \text{ eV}$ and ${4.93\times 10}^{16} \; { \text{m}}^{-3}$, which is consistent with the values reported for low pressure discharges^19^. Peak electron density occurs at $15\; \text{mm}$ insulator thickness.

**Figure 6:**
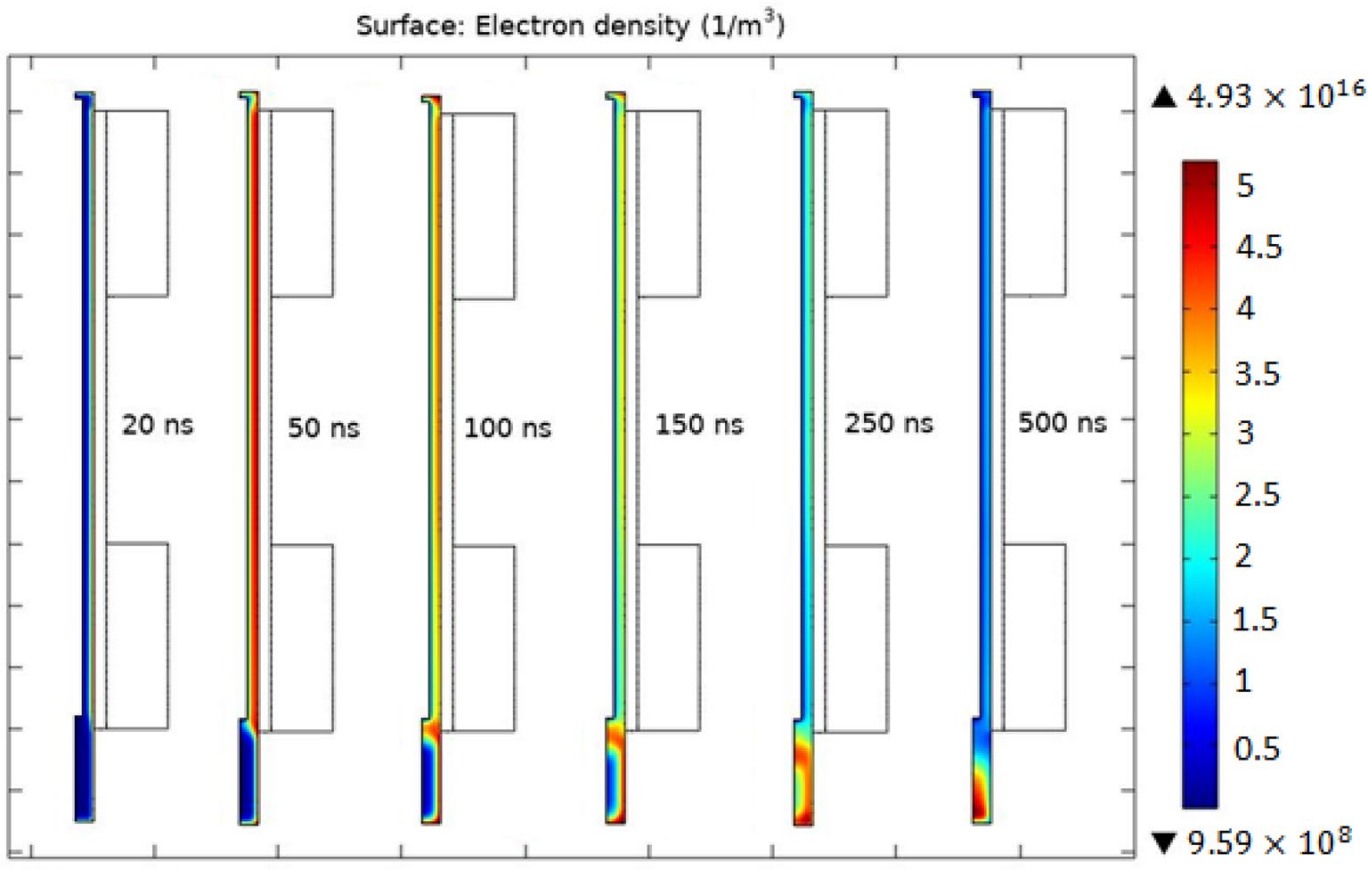
$JDBD$ propagation sequences out of the tube.

Figure 6 shows the jet propagation sequence of a large-scale plume forced into the open air in an unbounded region out of the tube. It has been theoretically proven that the proposed configuration is practical and ultimately an experimental set up is presented in the next section.

### *JDBD* architecture

**Figure 7:**
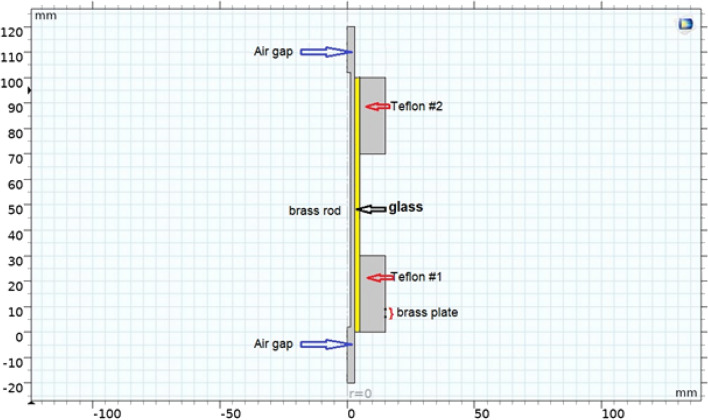
Schematic diagram of $JDBD$ experimental setup.

$Practical \; design \; task$ Of $JDBD$ is to generate large scale, $a \;  few \; cm$, and a stable plasma jet that is extended radially/axially in the open atmospheric air with multiple configurations. Simulation-based $JDBD$ with design parameters being: brass rod $100\; \text{mm}$ length and $2.5\; \text{mm}$ in diameter, glass insulator $100\; \text{mm}$ length and $10\; \text{mm}$ in diameter, Teflon $30\; \text{mm}$ length and $10\; \text{mm}$ thickness, brass plate $3\; \text{mm}$ length and $15\; \text{mm}$ thickness, is shown in Fig. 7. Minimum electric potential to ignite the discharge 10 kV at the optimum frequency $23 \; \text{kHz}$. The power supply was PVM500 with a maximum output $40 {kV}_{pk-pk}$, $18\; \text{mA}$, maximum output power of 300 W, and frequency of 20–70 kHz. High voltage probe was Tektronix P6015A with a ground-referenced $100 \; \text{M}\Omega$, 3.0 pF high voltage probe with $1000X$ attenuation. The Tektronix oscilloscope features are $200 \; \text{MHz}$ Bandwidth, and sample rate of $2.0 \; \text{GS/s}$.

$One{\text{-k}}\Omega$ resistor was placed in series with the groundand the voltage measured across the resistor was used to calculate the current by $Ohm$ law.

Typical voltage and current characteristic signal of the non-thermal plasma suggest that the average power consumed in the plasma is expressed by ${P}_{avg}={T}^{-1}{\int }_{0}^{T}I\left(t\right)V\left(t\right)dt$, where $T$ is the period.

**Figure 8:**
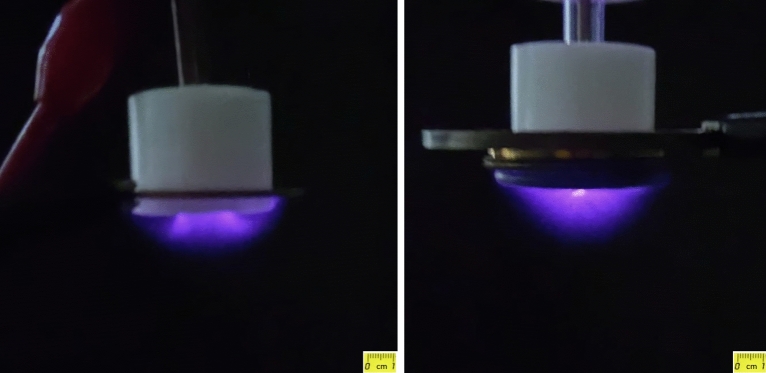
Large-scale $JDBD$ plasma: hollow (left) and solid (right) central electrode.

Recorded images of two $JDBD$ configurations among several designs are presented in Fig. 8 and displays extended/stable plasma jet in an unbound area in front of the tube with $a \;  few \; cm$ dimensions. This can be extended to a $10\times  10 \; {\text{cm}}^{2}$ uniform and stable non-thermal atmospheric air plasma source which is going to be discussed in great detail in the next paper.

**Figure 9:**
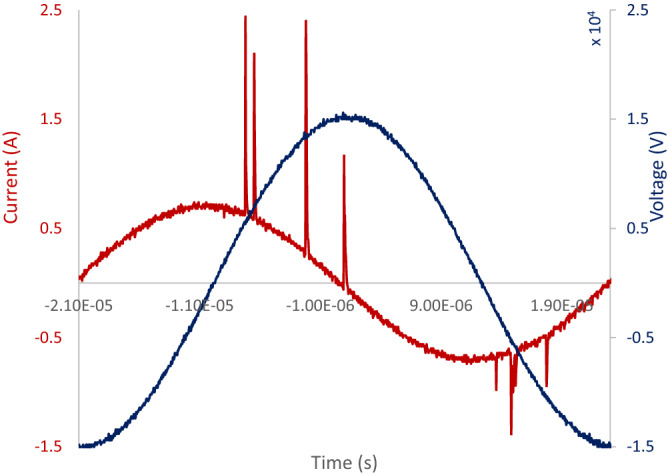
$JDBD$ current and voltage oscillograms.

The discharge characteristic is different for each cycle of the $AC$ voltage. Initially the anode connected to the high voltage behaves as an electron source at a negative cycle. This is featured as a dense and uniform discharge, which defines the current waveform. On the other hand, dielectric barrier (known as virtual electrode) acts as an electron source at the positive cycle of $AC$ voltage, which is characterized by high amplitude spikes in the current waveform with inconsistent temporal occurrence, Fig. 9. The discharge in the positive cycle is related to the filamentary structure and streamers, so that the localized ionization waves predominantly move from high voltage electrode to the virtual electrode through inconsistent propagation channels extended toward the dielectric barrier surface. The time scale of the filamentary discharges is $few \; hundreds \; of \; nanoseconds$, whereas the following streamers extend further with time. Discharge instabilities, just as contraction, result in the transition of the plasma structure from being diffuse to the filament configuration. Such transition is responsible for induced shift in the glow-to-arc discharge. The electron density and temperature are higher in the filaments than the diffuse discharges. In a homogenous plasma, both the electrons, left over from the previous discharges, and metastable atoms, are being present between the pulses and make the breakdown possible under lower electric fields. Dielectric surface sets off the required abundance of seed electrons and metastable particles^20^.

**Figure 10:**
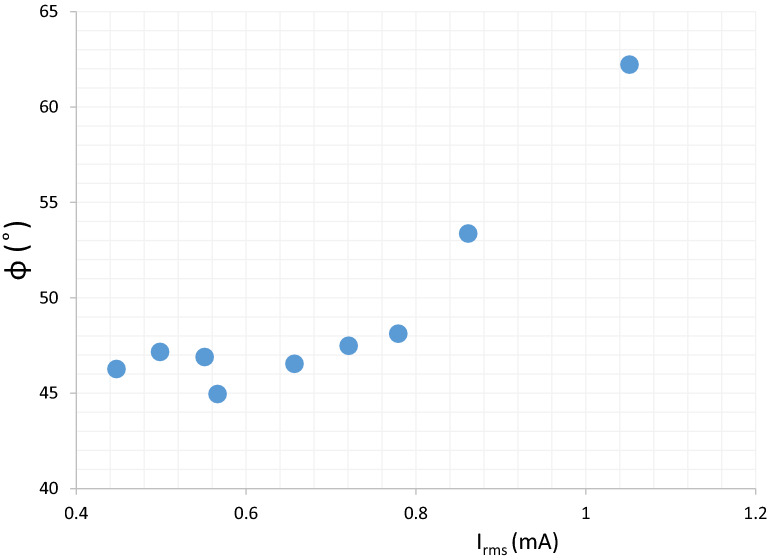
Phase angle representing capacitive nature of $JDBD$.

Power in the plasma dissipates through ohmic losses governed by the electron-neutral collisions. The plasma inductance is determined by the electron quantities opposing the electric field variations^21^. Phase shift is a measuring procedure to probe the plasma state, observe the impedance of the circuit, and look into the circuit behavior as a whole. Figure 10 shows phase angle versus ${I}_{rms}$ for the overall measured circuit including plasma. For the phase angle approaching zero, the discharge becomes more resistive with a proofing higher power.

Plasma acts as an additional nonlinear load into the circuit. When plasma is not formed, phase difference between voltage and current waveforms is about $\pi /2$, and the impedance is practically capacitive, and the voltage waveform lags current. In contrast, once the plasma is formed, phase difference decreases and the values for voltage and current changes.

Capacitive reactance builds up as a result of extended electrodes gap, corresponding to the maximum sheath/space charge width. At higher electric fields, the ion density increases due to the high electron concentration, representing a collisional heating source thus the power is escalated.

Figure 10 shows that phase shift increases by ${I}_{rms}$, thus in $JDBD$, the capacitive plasma generates more power than the resistive plasma due to the higher probability of the space charge formation^22^. At lower current, the phase shift is uniform and cluster about $\pi /4$, whereas at $0.8\; \text{mA}$, capacitive nature of the $JDBD$ steps up to $\pi /3$. This implies two distinct states of $JDBD$ operation indicating the mode-swap at $0.8\; \text{mA}$.

**Figure 11:**
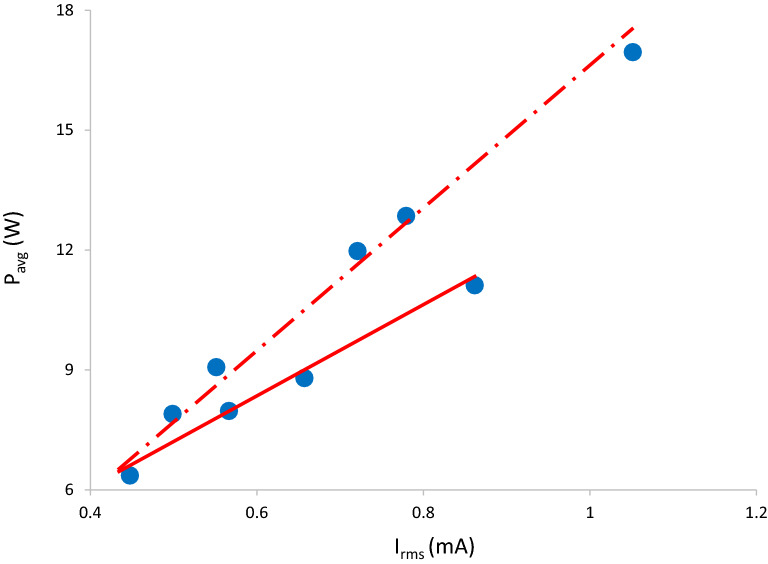
Measured power of typical plasma jets (solid) and $JDBDs$ (dash-dotted).

Figure 11 shows the measured power as a function of ${I}_{rms}$ for $JDBD$ at atmospheric air, in which the power dissipation rises with current. Time-averaged power is obtained by the circuit components including voltage, current, and the phase shift. In the reactive $JDBD$ scheme, at larger $Cosine \; gradients$, the phase angle acts as an effective control parameter for power. Solid-line in this figure represents the experimental data obtained from the plasma jet devices, with a typical electrode/insulator assembly as in Ref.^23^, and compared to a few designed $JDBD$ configurations, dashed-line.

## Conclusions

$JDBD$ Configuration resolves both small-scale jet and bound DBD plasma limitations through optimizing the electric field by an efficient insulation as a key design parameter. The current density with a sudden steep and a constant value running at peak electric fields, backs up the $JDBD$ geometry. Mean electron temperature and maximum electron density was computed to be about $2.1 \; \mathrm{eV}$ and ${4.93\times 10}^{16} \;{ \mathrm{m}}^{-3}$ at $15\; \mathrm{mm}$ insulator thickness. In $JDBD$, the plasma generates more power than the conventional plasma due to the higher probability of the space charge formation. At lower current, the phase shift is uniform and cluster about $\pi /4$, whereas at a definite higher current, the $JDBD$ phase steps up to $\pi /3$ that implies mode-swap operation.

## Data Availability

The data that support the findings of this study are available within the article.
